# S61. THE ASSOCIATION OF VERBAL LEARNING DEFICITS WITH AGE AND SYMPTOMS IN SCHIZOPHRENIA

**DOI:** 10.1093/schbul/sby018.848

**Published:** 2018-04-01

**Authors:** Dimitrios Kontis, Alexandra Giannakopoulou, Eirini Theochari, Angeliki Andreopoulou, Spyridoula Vassilouli, Dimitra Giannakopoulou, Eleni Siettou, Eleftheria Tsaltas

**Affiliations:** 1 Psychiatric Hospital of Attica; 2 Athens University Medical School

## Abstract

**Background:**

The relationship of age and symptoms with the performance on verbal learning and memory tasks in schizophrenia could provide useful information for optimizing and individualizing the efforts to remediate the cognitive impairments of patients.

**Methods:**

During a cross-sectional study, 97 medicated and stabilized patients with chronic schizophrenia (61 males and 36 females, mean age=43.74 years, standard deviation-SD=11.59), which were consecutively referred to our Unit, were assessed using the Hopkins Verbal Learning Test (HVLT) and the Positive and Negative Syndrome Scale (PANSS). A linear regression analysis was conducted in order to investigate the effect of symptoms and age on HVLT performance.

**Results:**

Increased age and total PANSS symptoms were associated with worse total recall (raw scores) (B=-0.109. 95% confidence interval-C.I.- =-0.18, -0.038, t=-3.038, df=90 p=0.003 and B=-0.053, 95%CI=-0.097, -0.008, t=-2.356, df=90, p=0.021, respectively). The effect of symptoms on HVLT total recall was significant for positive (B=-0.166, 95%CI=-0.316, -0.015, t=-2.189, df=90, p=0.031), negative (B=-0.167, 95%CI=-0.279, -0.054, t=-2.949, df=90, p=0.004), but not for general psychopathology symptoms (B=-0.05, 95%CI=-0.129, 0.03, t=-1.247, df=90, p=0.216). Further analyses revealed the significant negative correlations of total symptoms with the performance in immediate recall during the first HVLT trial (B=-0.021, 95% CI=-0.036, -0.005, df=89, p=0.011), and age during the second (B=-0.046, 95%CI=-0.076,-0.017, p=0.003) and third (B=-0.048, 95%CI=-0.083, -0.014, df=89, p=0.007) HVLT immediate recall trials. Both total symptoms and age were significantly negatively correlated with the performance in recognition discrimination (raw scores) (symptoms: B=-0.199, 95%CI=-0.363, -0.035, df=87, t=-2.415, p=0.017 and age: B=-0.357, 95%CI=-0.617, -0.098, df=87, t=-2.737, p=0.008). We failed to find any significant correlation between either age or symptoms with delayed recall.

**Discussion:**

Age and symptoms are associated with immediate verbal learning and memory impairments but not with deficits in verbal delayed recall in schizophrenia. The effects of medication remain to be explored in future analyses. Cognitive remediation programmes against verbal learning deficits in individuals with schizophrenia should take into account their age as well as their symptomatology.

